# Inter-domain Communication of Human Cystathionine β-Synthase: STRUCTURAL BASIS OF S-ADENOSYL-l-METHIONINE ACTIVATION*

**DOI:** 10.1074/jbc.M114.610782

**Published:** 2014-10-21

**Authors:** Thomas J. McCorvie, Jolanta Kopec, Suk-Joon Hyung, Fiona Fitzpatrick, Xidong Feng, Daniel Termine, Claire Strain-Damerell, Melanie Vollmar, James Fleming, Jay M. Janz, Christine Bulawa, Wyatt W. Yue

**Affiliations:** From the ‡ Structural Genomics Consortium, Nuffield Department of Clinical Medicine, University of Oxford, Oxford OX3 7DQ, United Kingdom,; § Worldwide Research and Development, Pfizer Inc., Groton, Connecticut 06340, and; the ¶ Pfizer Rare Disease Research Unit, Worldwide Research and Development, Pfizer Inc., Cambridge, Massachusetts 02140

**Keywords:** Allosteric Regulation, Conformational Change, Crystallography, Enzyme, Mass Spectrometry (MS), S-adenosyl-l-methionine (AdoMet), Activation, Cystathionine β-Synthase, SAM

## Abstract

Cystathionine β-synthase (CBS) is a key enzyme in sulfur metabolism, and its inherited deficiency causes homocystinuria. Mammalian CBS is modulated by the binding of *S*-adenosyl-l-methionine (AdoMet) to its regulatory domain, which activates its catalytic domain. To investigate the underlying mechanism, we performed x-ray crystallography, mutagenesis, and mass spectrometry (MS) on human CBS. The 1.7 Å structure of a AdoMet-bound CBS regulatory domain shows one AdoMet molecule per monomer, at the interface between two constituent modules (*CBS-1, CBS-2*). AdoMet binding is accompanied by a reorientation between the two modules, relative to the AdoMet-free basal state, to form interactions with AdoMet via residues verified by mutagenesis to be important for AdoMet binding (Phe^443^, Asp^444^, Gln^445^, and Asp^538^) and for AdoMet-driven inter-domain communication (Phe^443^, Asp^538^). The observed structural change is further supported by ion mobility MS, showing that as-purified CBS exists in two conformational populations, which converged to one in the presence of AdoMet. We therefore propose that AdoMet-induced conformational change alters the interface and arrangement between the catalytic and regulatory domains within the CBS oligomer, thereby increasing the accessibility of the enzyme active site for catalysis.

## Teaser

**Background:** Human cystathionine β-synthase (hCBS) is activated by *S*-adenosyl-l-methionine (AdoMet).

**Results:** We presented structural and solution evidence that AdoMet binding to hCBS regulatory domain causes conformational rearrangement to the protein.

**Conclusion:** AdoMet activates hCBS by altering the interface and arrangement between its catalytic and regulatory domains.

**Significance:** Our data identified the key residues for AdoMet binding and provide a mechanism for allosteric activation.

## Introduction

Cystathionine β-synthase (CBS,^3^ EC 4.2.1.22) plays an important role in methionine and sulfur metabolism, and is a unique heme and pyridoxal 5′-phosphate (PLP)-containing enzyme (1, 2). Its canonical role is the condensation of serine and l-homocysteine (Hcy) to form cystathionine, which can be further metabolized to cysteine or glutathione. Recently, CBS has been identified as a major contributor to hydrogen sulfide production, an important secondary messenger (3). Inherited mutations on the *cbs* gene lead to classical homocystinuria (OMIM 236200), an autosomal recessive disorder of methionine metabolism characterized by deficient CBS enzyme and elevated plasma Hcy level. To date more than 160 *cbs* disease alleles, predominantly missense mutations (>87%), have been identified with the most prevalent p.I278T allele accounting for ∼25% of patients (4). Clinical presentations, likely due to Hcy accumulation in various tissues, are associated with abnormalities in the eye, skeleton, vascular system, and central nervous system (5). For some disease alleles, misfolding of CBS mutant enzymes is proposed to contribute to disease pathogenicity (6).

**FIGURE 1.**
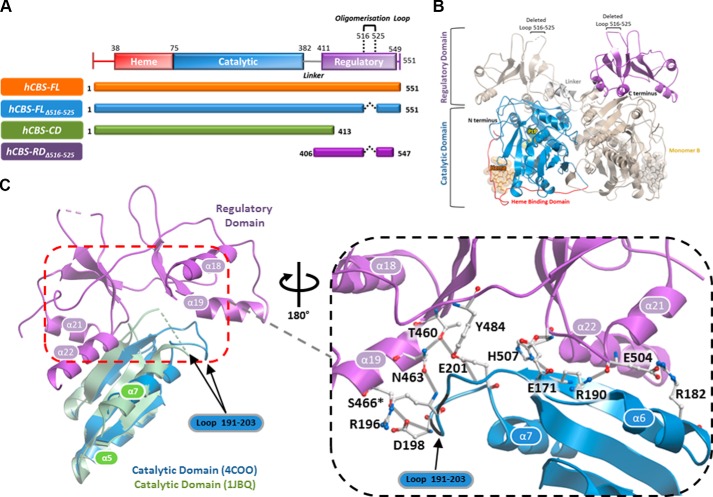
**Structure and domain organization of hCBS.** *A*, overview of hCBS constructs used in this study. *Black dotted lines* indicate the removal of a disordered loop (aa 516–525) in the constructs hCBS-FL_Δ516–525_ and hCBS-RD_Δ516–525_. *B*, structure of hCBS-FL_Δ516–525_ showing the domain-swapped dimer (monomer A, *red*, *blue*, and *purple*; monomer B, *gold*). *C*, structural overlay of hCBS-FL_Δ516–525_ in the AdoMet-free state (PDB 4COO; *purple*, regulatory domain; *blue*, catalytic domain) with the activated catalytic domain alone (PDB 1JBQ, *green*). *Inset*, interactions between the regulatory domain (*purple*) and active site loops in the catalytic domain (*blue*) of hCBS-FL_Δ516–525_, including the hydrogen bonds of Ser^466^ with Asp^198^ and Arg^196^ of the active site loop.

CBS adopts a three-domain structure encompassing the N-terminal heme binding, central catalytic, and C-terminal regulatory domains (see Fig. 1*A*). Its functional oligomeric state is believed to be a tetramer, although higher order species are known to exist (4, 7–9). In mammals, the activity of CBS catalytic domain is increased up to 5-fold by the binding of *S*-adenosyl-l-methionine (AdoMet) to the regulatory domain (7), an ∼140-amino acid stretch harboring a tandem module (*CBS-1, CBS-2*, collectively known as the Bateman motif (10)) found in a number of proteins (11). Until recently, the only available structural information was from the dimeric heme binding and catalytic domains of human CBS (hCBS) (2, 12), as well as the *Drosophila melanogaster* orthologue (dmCBS) with a regulatory domain that is not responsive to AdoMet activation (13). The hCBS regulatory domain in its AdoMet-free state was later revealed in the context of a full-length dimeric structure (14). This was engineered with a loop deletion (aa 516–525), identified by homology modeling and sequence alignment with dmCBS to be important in tetramer formation and to facilitate crystal formation. The hCBS structure reveals a domain-swapped arrangement, not seen in dmCBS, between the two regulatory domains and catalytic domains within the homodimer, covering the entrance of the active sites of the enzyme. However, the manner in which AdoMet binds to the regulatory domain and its impact on the interaction between regulatory and catalytic domains remain unknown. Biophysical studies have suggested two types of AdoMet binding sites on hCBS (referred to as sites S1 and S2 (15)), purportedly with different effects on the protein (16). The stoichiometry of AdoMet binding to CBS is also not clear, with proposals ranging from four AdoMet molecules (4, 8, 17), to more recently six per tetramer (16).

In the absence of a molecular understanding of how AdoMet binds to and regulates CBS activity, we report here the 1.7 Å resolution crystal structure of an hCBS regulatory domain in complex with AdoMet. This structure depicts a dimeric arrangement of the regulatory domain that agrees with a 3.6 Å AdoMet-bound structure of an hCBS full-length variant, reported during the preparation of this manuscript (18). Our high resolution structural data further provide atomic details of AdoMet binding interactions and reveal, in conjunction with our solution-based biophysical analysis, significant conformational changes within the CBS modules upon binding AdoMet. These conformational changes are necessary for AdoMet binding specificity and provide insight into how the regulatory and catalytic domains interface to enable AdoMet modulation of catalytic activity.

## EXPERIMENTAL PROCEDURES

### 

#### 

##### Cloning, Expression, and Purification of hCBS Proteins

Initially, a DNA fragment encoding hCBS-FL (aa 1–551; IMAGE clone: 3357140) was subcloned into the pNIC28-Bsa4 vector (GenBank^TM^ accession EF198106), incorporating an N-terminal tobacco etch virus-cleavable His_6_ tag. This was subsequently used to create hCBS-CD (aa 1–413), as well as hCBS-FL_Δ516–525_ with an engineered loop deletion, which was in turn used to generate hCBS-RD_Δ516–525_ (aa 406–547). hCBS-FL with a non-cleavable C-terminal His_6_ tag was obtained by subcloning into pNIC-CH incorporating a non-cleavable C-terminal His_6_ tag (GenBank accession EF199843). Alanine substitutions (p.F443A, p.D444A, p.Q445A, p.H507A, p.T535A & p.D538A) were constructed using the QuikChange site-directed mutagenesis kit (Stratagene) on hCBS-FL. All plasmids were transformed into *Escherichia coli* BL21(DE3)-R3-pRARE2 cells and expressed with induction by 0.1 mm isopropyl-β-d-thiogalactopyranoside overnight at 18 °C. hCBS-FL, hCBS-FL_Δ516–525_, hCBS-CD, and hCBS-RD_Δ516–525_ proteins were purified by affinity chromatography (Talon; Clontech) and gel filtration (Superdex 200; GE Healthcare) followed by His_6_ tag removal overnight and further purification by reverse affinity (nickel-nitrilotriacetic acid; Qiagen) and anion exchange (HiTrap Q; GE Healthcare). Purification of hCBS-FL and alanine mutants was carried out as described (19) except that the anion exchange step used a HiTrap Q column, and protein was further purified by gel filtration (Superose 6 prep; GE Healthcare).

##### Crystallization and Structure Determination of hCBS-FL_Δ516–525_ and hCBS-RD_Δ516–525_

Crystals were grown by vapor diffusion at 4 °C, involving sitting drops that comprise 150 μl of hCBS-FL_Δ516–525_ (10.8 mg/ml) mixed with 150-μl reservoir (18% PEG8000, 0.1 m cacodylate, pH 6.8, and 0.2 m calcium acetate) or sitting drops that contain 50 μl hCBS-RD_Δ516–525_ (5 mg/ml) mixed with 100-μl reservoir (36% PEG550MME, 0.1 m Tris, pH 7.5, and 0.2 m calcium chloride). Crystals were cryoprotected using 25% ethylene glycol and flash-cooled in liquid nitrogen. All diffraction data were collected at the Diamond Light Source and processed with the CCP4 suite (20). The hCBS-FL_Δ516–525_ structure was solved by molecular replacement using the hCBS catalytic domain structure (Protein Data Bank (PDB) 1JBQ) as a search model in PHASER (21). Iterative cycles of restrained refinement and manual model building were performed using COOT (22) and REFMAC5 (23). The structure of hCBS-RD_Δ516–525_ was solved by single anomalous dispersion phasing from a mercury-derivatized crystal (soaked with 10 mm ethyl mercury thiosalicylate in reservoir solution for 10 min). Mercury atoms were located using ShelxD and subsequently used to calculate initial phases in ShelxE (24). The initial model was built using BUCCANEER followed by iterative cycles of restrained refinement and manual model building in COOT (22) and REFMAC5 (23).

##### Differential Scanning Fluorometry, Limited Proteolysis, and UV Spectra

The various CBS proteins were assayed for shifts in melting temperature caused by ligand binding in a 96-well PCR plate using an Mx3005p RT-PCR machine (Stratagene) with excitation and emission filters of 492 and 610 nm respectively. Each well (20 μl) consisted of protein (5 μm in 10 mm HEPES, pH 7.5, 150 mm NaCl), SYPRO-Orange (Invitrogen, diluted 1000-fold of the manufacturer's stock), and various concentrations of ligand. Fluorescence intensities were measured from 25 to 96 °C with a ramp rate of 1 °C/min. The *T_m_* was determined by plotting the intensity as a function of temperature and fitting the curve to a Boltzmann equation (25, 26). Temperature shifts, Δ*T_m_*, for each ligand were also determined as described (25, 26). AC_50_ values (half-maximal effective ligand concentration) and final graphs were generated using GraphPad Prism (v.5.01; Graph-Pad Software).

Limited proteolysis with thermolysin was carried out as described in 20 mm Tris-HCl (pH 8.0) with 10 mm CaCl_2_ at room temperature (27). Remaining intact protein for different time points was determined by a combination of SDS-PAGE and ImageJ software (rsbweb.nih.gov/ij/), which was used to determine band intensities. Rates of proteolysis (*k_p_*) were determined by plotting the percentage of remaining intact protein against time and fitting to the following non-linear equation

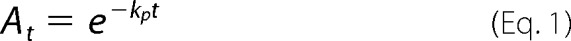
 where *A_t_* is the percentage of remaining intact protein at time *t* and *k_p_* is the rate of proteolysis. Serine and AdoMet were added to assays at a final concentration of 1 mm when appropriate. UV-visible spectra were determined using a NanoDrop 2000 (Thermo Scientific) in 50 mm HEPES, pH 7.5, 500 mm NaCl, 0.5 mm tris(2-carboxyethyl)phosphine, 5% glycerol.

##### Mass Spectrometry

Ion mobility MS spectra were recorded on a hybrid quadrupole IM-ToF instrument (Waters, Milford, MA). For ion mobility mass spectrometry (IM-MS), 20 μl of purified hCBS-FL (1.2 mg/ml) was exchanged into 200 mm ammonium acetate buffer (pH 7.5) and diluted to a final concentration of 20 μm monomer, and AdoMet was added at 20 μm when appropriate. 2-μl aliquots were electrosprayed from gold-coated borosilicate capillaries Thermo Scientific). The instrument conditions were optimized to preserve non-covalent interactions (28). The instrument was operated with backing pressure at 9.18 millibars and traveling wave IM separator pressurized with nitrogen and helium at 3.44 × 10^−2^ millibars. IM separation was made using following sets of traveling wave velocity and traveling wave height parameters: 600 m/s, 30 V; 700 m/s, 35 V; 700 m/s, 30 V. Mass spectra were analyzed using MassLynx 4.1.1 and Driftscope 2.3 software (Waters). Collision cross-section (ohms) measurements were externally calibrated using a database of reference values in helium, with values for peptides that bracket the collision cross-section and IM values of the unknown ions (29).

## RESULTS

### 

#### 

##### Crystallization of hCBS Proteins with and without AdoMet

Our initial attempts to crystallize human CBS adopted the multi-construct approach (Fig. 1*A*), surveying different construct boundaries including the full-length protein and then attaching the His_6_ tag to either the N terminus or the C terminus. The resultant proteins are largely of low soluble yield, highly degraded during purification, and recalcitrant to crystallization. We next engineered a loop deletion in the regulatory domain of hCBS full-length (aa 516–525; protein hereafter referred to as hCBS-FL_Δ516–525_), as per reported approach (14), and observed improvement in soluble protein yield (10-fold) and decreased degradation, as compared with the “loop-intact” full-length protein (hCBS-FL). Importantly, hCBS-FL_Δ516–525_ crystallized, and its structure in the AdoMet-free, heme-, and PLP-bound form was determined to 2.0 Å resolution (Table 1). The hCBS-FL_Δ516–525_ structure reveals a domain-swapped dimer where the C-terminal regulatory domain of one subunit is atop the N-terminal catalytic domain of the other, and *vice versa* (Fig. 1*B*). In the regulatory domain, two cavities (sites S1 and S2) are found at opposite faces of the *CBS-1*:*CBS-2* modular interface, with S2 being more solvent-accessible than S1. The S2 face is also where a number of inter-subunit contacts between regulatory and catalytic domains are found (Fig. 1*C*). Specifically, *CBS-1* and *CBS-2* make a number of hydrogen bonds with the active site loops 191–203 and 170–175, respectively. These contacts are likely to be important in the regulatory the ability of the domain to modulate the activity of the catalytic domain.

**TABLE 1:** **Crystallography refinement statistics** Data for highest resolution shell are shown in parentheses. Hg, mercury; Anom, anomaly.

	hCBS-FL_Δ516–525_	hCBS-RD_Δ516–525 (native)_	hCBS-RD_Δ516–525 (Hg derivative)_
**Overall description**			
PDB code	4COO	4UUU	
Ligands bound	PLP, HEM	AdoMet	AdoMet, thiomersal

**Data collection**			
Beamline	Diamond I04	Diamond I02	Diamond I04–1
Wavelength (Å)	0.9795	0.97949	0.9207
Unit cell parameters (Å)	70.26 116.19 167.99	39.85 78.65 90.80	40.08 80.06 81.22
α = β = γ (°)	90.00	90.00	90.00
Space group	P2_1_2_1_2_1_	P2_1_2_1_2_1_	P2_1_2_1_2_1_
Resolution range (Å)	48.89–2.00 (2.03–2.00)	39.09–1.71 (1.71–1.76)	40.08–1.75 (1.80–1.75)
Observed/Unique reflections	418,361/93,295 (20,805/4527)	136,884/31,258 (9834/2207)	391,150/30,570 (24,675/2203)
*R*_merge_ (%)	0.069 (0.85)	0.055 (0.491)	0.071 (0.733)
*I*/σ(*I*)	15.1 (2.0)	12.1 (2.1)	20.6 (3.5)
Completeness	99.8 (99.8)	99.1 (97.0)	Anom 99.8 (99.7)
Multiplicity	4.5 (4.6)	4.4 (4.5)	Anom 6.8 (5.8)
Anomalous correlation			0.512 (0.021)
Anomalous slope			1.216

**Refinement**			
*R*_cryst_ (%)	17.52	15.91	
*R*_free_ (%)	20.07	19.86	
Wilson *B* factor (Å^2^)	32.6	21.57	
Average total *B* factor (Å^2^)	43.81	19.342	
Average ligand *B* factor (Å^2^)	PLP, 38.01; HEM, 47.77	AdoMet, 20.04	
Ligand occupancy	1	1	
r.m.s.d. bond length (Å)	0.0099	0.0165	
r.m.s.d. bond angle (°)	1.4296	1.7443	
Ramachandran outliers (%)	1 (0.36%)	0 (0.0%)	
Ramachandran favored (%)	274 (98.55%)	959 (97.86%)	

To understand the structural consequence of AdoMet binding, we attempted to determine the AdoMet-bound hCBS-FL_Δ516–525_ structure by co-crystallization or soaking of AdoMet-free crystals with the ligand. These were not successful, although hCBS-FL_Δ516–525_ binds AdoMet in solution as with hCBS-FL (14). To this end, we constructed the hCBS regulatory domain (aa 406–547) as a standalone N-terminal His_6_-tagged protein, with the aa 516–525 loop either removed (hCBS-RD_Δ516–525_) or intact (hCBS-RD), for structural studies. Only hCBS-RD_Δ516–525_, but not hCBS-RD, was soluble and yielded crystals in the presence of AdoMet, diffracting to 1.7 Å resolution (Table 1). To our surprise, the structure of AdoMet co-crystallized hCBS-RD_Δ516–525_ could not be solved by molecular replacement using an AdoMet-free regulatory domain model extracted from our hCBS-FL_Δ516–525_ structure, but instead by single-wavelength anomalous dispersion with a mercury-derivatized crystal. This suggested the possibility of a conformational change within this domain upon AdoMet binding.

##### Conformational Change Mediated by One Bound AdoMet per Regulatory Domain

**FIGURE 2.**
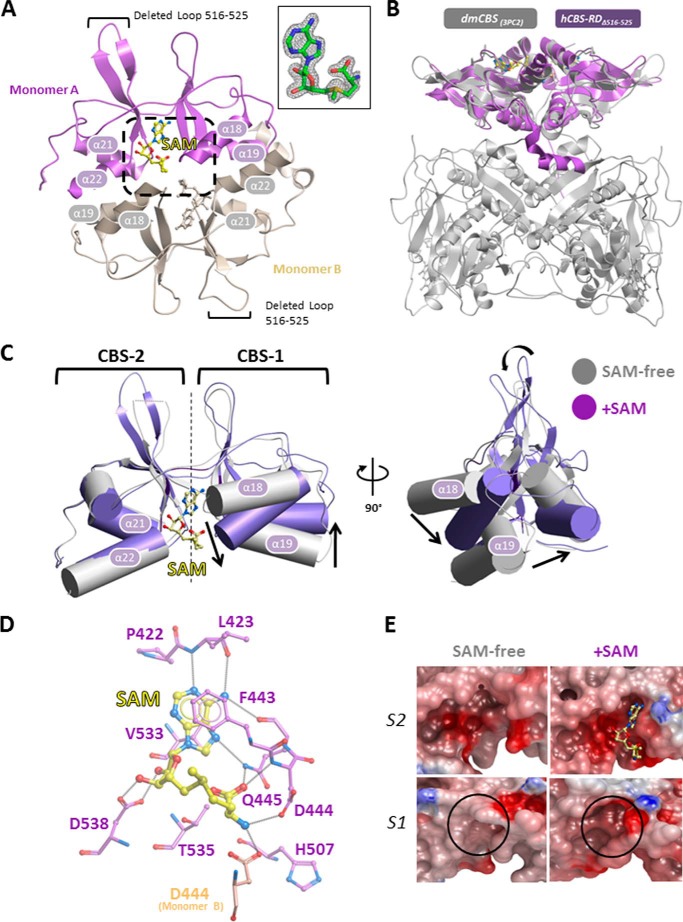
**AdoMet binding in the regulatory domain is accompanied by a conformational change.** *A*, structure of hCBS-RD_Δ516–525_ bound with AdoMet reveals a dimeric arrangement. *Inset*, 2*F^o^* − *F^c^* electron density of AdoMet. *B*, structure superposition of hCBS-RD_Δ516–525_ (this study, *purple*) and full-length dmCBS (PDB 3PC2, *gray*). The regulatory domains of the two structures are structurally homologous and reveal a similar dimeric arrangement. *C*, structural overlay of the regulatory domain from AdoMet-free (*SAM-free*) hCBS-FL_Δ516–525_ (*gray*) with that of AdoMet-bound hCBS-RD_Δ516–525_ (*purple*), highlighting the relative arrangement of CBS-1 module with respect to CBS-2. *D*, stick representation of AdoMet binding residues in the regulatory domain. AdoMet binds to the S2 site via a number of key residues, including Asp^444^ from the opposing dimeric subunit. *E*, surface electrostatic representation of the S2 and S1 sites in the AdoMet-free and AdoMet-bound structures.

The hCBS-RD_Δ516–525_ crystal asymmetric unit contains a “head-to-tail” dimer mediated by a two-fold non-crystallographic symmetry (C_α_-r.m.s.d. of 0.241 Å between the two subunits) (Fig. 2*A*). This is an arrangement previously seen in some CBS module-containing proteins (30) and is highly homologous to the arrangement of the domain in dmCBS structure (C_α_-r.m.s.d. = 1.22 Å; Fig. 2*B*) (13). There is unambiguous electron density for one AdoMet molecule per regulatory domain, bound at the cleft between *CBS-1* and *CBS-2* modules (Fig. 2*A*, *inset*). Our high-resolution hCBS-RD_Δ516–525_ structure agrees with the regulatory domain arrangement, as well as AdoMet binding region and stoichiometry, from those of an hCBS full-length structure bound to AdoMet (C_α_-r.m.s.d. = 0.33 Å for 130 aligned atoms), recently reported at 3.6 Å resolution, that incorporated the Δ516–525 loop deletion and an E201S mutation at the catalytic-regulatory domain interface (hereafter referenced as hCBS-FL_Δ516–525,E201S_) (18).

A structural overlay of our AdoMet-bound hCBS-RD_Δ516–525_ with the AdoMet-free equivalent (extracted from hCBS-FL_Δ516–525_ coordinates) shows that they are only moderately superimposable (C_α_-r.m.s.d. 3.0 Å), which would be a likely explanation for the unsuccessful molecular replacement solution. Although the topologies of *CBS-1* and *CBS-2* modules are largely unchanged between the two structures, AdoMet binding is accompanied by an ∼33° rotational rearrangement between *CBS-1* and *CBS-2*, mediated by a bending of the inter-module linkers (aa 422–423, 480–487) that act as the hinge (Fig. 2*C*). Relative to *CBS-2*, the secondary structure elements of *CBS-1* (strands β12-β13, helices α18 and α19) are displaced by as much as 8 Å from their AdoMet-free positions, thereby translocating residues (*e.g.* Phe^443^-Asp^444^-Gln^445^, FDQ motif) into bonding distances with the ligand (Fig. 2*D*).

AdoMet adopts a folded configuration with regard to its methionyl moiety, as observed in several ligand-bound CBS module structures (15, 30). The AdoMet pocket in hCBS, occupying the S2 site (Fig. 2*E*), is formed on one side by the ^443^FDQ^445^ motif in *CBS-1*, on the other side by the ^532^GVVTAID^538^ motif in *CBS-2*, and at its end by the *CBS-1*→*CBS-2* linker region (aa 414–423) (Fig. 2*D*). The FDQ motif contributes an aromatic stacking interaction with the AdoMet adenine ring (Phe^443^), as well as hydrogen bonds with the methionyl nitrogen (Asp^444^) and carboxyl (Gln^445^) groups. In addition, the methionyl nitrogen is further hydrogen-bonded to Asp^444^ from the opposite dimeric subunit. The GVVTAID motif, highly conserved among CBS module-containing proteins, contributes a bifurcated hydrogen bond to the AdoMet ribosyl oxygens (Asp^538^) and a hydrogen bond to the methionyl carboxyl (Thr^535^). In addition, Val^533^, along with Pro^422^ and Leu^423^ from the *CBS-1*→*CBS-2* linker, contribute hydrophobic interactions with the AdoMet adenine ring (Fig. 2*D*).

##### Regulatory Domain Dynamics Revealed by DSF and Limited Proteolysis

**FIGURE 3.**
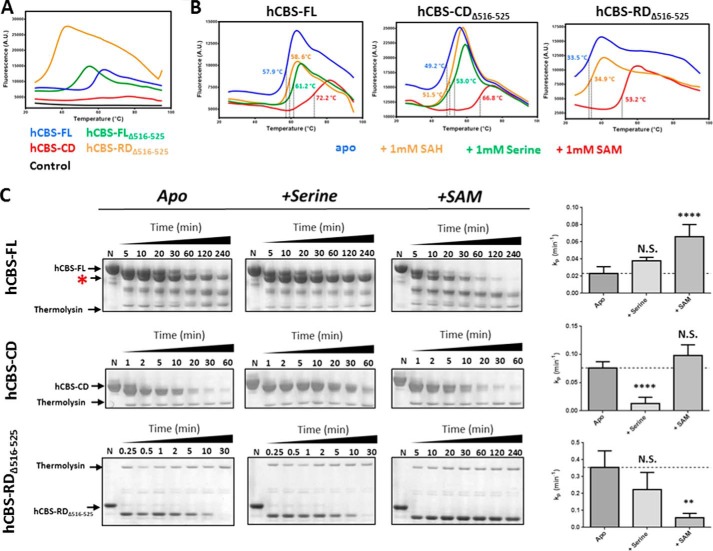
**Biophysical studies to probe ligand binding responses of various hCBS constructs.** *A*, representative thermal unfolding curves for the four hCBS constructs in their as-purified forms. *a.u.*, arbitrary units. *B*, representative thermal unfolding curves for hCBS-FL, hCBS-FL_Δ516–525_, and hCBS-RD_Δ516–525_ in the presence of different ligands (serine, *S*-adenosyl-l-homocysteine (*SAH*), and AdoMet (*SAM*) at 1 mm each). *C*, thermolysin-limited proteolysis of hCBS-FL, hCBS-CD, and hCBS-RD_Δ516–525_ demonstrates the response of each construct to AdoMet and serine. Representative gels are shown (*left*), accompanied by plots of the determined rate of proteolysis (*k_p_*, *right*). For hCBS-FL, a band at ∼55 kDa (smaller than full-length but larger than the catalytic domain), which was protected in the presence of serine, is shown with *asterisk*. Rates of proteolysis are reported as the means and standard deviations from at least three independent experiments. *p* values were determined using two-tailed unpaired *t* test. *N.S.*, non-significant, *, *p* < 0.05, **, *p* < 0.01, ***, *p* < 0.001, ****, *p* < 0.0001.

To better understand how AdoMet binding modulates CBS function, we assessed the impact on thermal stability and global conformational dynamics of our various constructs (hCBS-FL, hCBS-FL_Δ516–525,_ hCBS-CD, hCBS-RD_Δ516–525_; Fig. 1*A*) using differential scanning fluorometry (DSF) and limited proteolysis, techniques applied previously to study full-length CBS (8, 27, 31). Initially, DSF was employed to determine the *T_m_* of various constructs in their as-purified form (Fig. 3*A*). This technique uses a fluorescent hydrophobic dye that binds to hydrophobic regions of proteins and thus allows the monitoring of unfolding against denaturation and the determination of the temperature (*T_m_*) at which a protein is half-denatured.

The *T_m_* of hCBS-FL (57.9 °C) agrees well with the value (56 °C) previously determined by differential scanning calorimetry (32). Both hCBS-FL_Δ516–525_ and hCBS-RD_Δ516–525_ exhibited a lower *T_m_* (49.2 and 33.5 °C) than hCBS-FL, likely an impact of removing loop 516–525 and the catalytic domain on the remainder of the polypeptide. These changes in *T_m_* suggest a level of communication between the two domains. Importantly hCBS-CD did not produce a discernible melting curve in DSF (Fig. 3*A*, *red*) (and was not used in subsequent DSF experiments), suggesting that the thermal unfolding event observed for the other constructs is attributable to the regulatory domain.

The effect of known CBS ligands on thermal stability of the three applicable constructs was then determined (Fig. 3*B*), with the rationale that target-specific ligands can stabilize a protein against thermal insult (26) and elicit a shift in *T_m_* values. All constructs exhibited a dose-dependent response to their respective ligands, which could be fitted to a Michaelis-Menten-like equation (Table 2) (26). We observed that constructs encompassing the regulatory domain (hCBS-FL, hCBS-FL_Δ516–525,_ hCBS-RD_Δ516–525_) were thermally stabilized by AdoMet (maximal shift in *T_m_*, Δ*T_m_*max = 16.3, 19.5, and 20.7 °C, respectively, at 1 mm ligand) and, to a much lesser extent by *S*-adenosyl-l-homocysteine, an AdoMet analogue (Δ*T_m_*max = 1.3, 4.3, and 5.4 °C, respectively). Constructs encompassing the catalytic domain (where serine is expected to bind), namely hCBS-FL and hCBS-FL_Δ516–525_ but not hCBS-RD_Δ516–525_, were thermally stabilized by serine (Δ*T_m_*max = 3.3 and 4.4 °C, respectively). With the observation that hCBS-CD does not elicit a response to DSF with or without ligands, the thermal stabilization seen with serine in hCBS-FL and hCBS-FL_Δ516–525_ is likely an effect communicated onto the regulatory domain.

**TABLE 2:** **Apparent AC_50_ and Δ*T_m_*max values of hCBS constructs applicable to DSF** SAH, *S*-adenosyl-l-homocysteine; ND, not determined.

Constructs	Serine		SAH		AdoMet	
	Apparent AC_50_	Apparent Δ*T_m_*max	Apparent AC_50_	Apparent Δ*T_m_*max	Apparent AC_50_	Apparent Δ*T_m_*max
	μ*m*	°*C*	μ*m*		μ*m*	°*C*
hCBS-FL	55.7 ± 6.6	3.3 ± 0.1	197.0 ± 65.8	1.3 ± 0.2	133.2 ± 17.7	16.3 ± 0.6
hCBS-FL_Δ516–525_	61.9 ± 7.0	4.4 ± 0.1	406.8 ± 79.7	4.3 ± 0.4	101.2 ± 18.5	19.5 ± 1.1
hCBS-RD_Δ516–525_	ND	ND	542.2 ± 62.1	5.4 ± 0.3	95.8 ± 16.6	20.7 ± 1.0

The response to AdoMet and serine by hCBS was also studied by limited proteolysis, a method that assesses the susceptibility of protein toward proteolytic cleavage (*e.g.* thermolysin). The presence of AdoMet increases the rate of thermolysin proteolysis (*k_p_*_,+AdoMet_/*k_p_*_,−AdoMet_) for hCBS-FL (by ∼2.9-fold), but not for hCBS-CD (Fig. 3*C*, *top versus middle*). This rate increase was previously postulated to reflect an allosteric mechanism through which AdoMet binding to hCBS regulatory domain relieves its steric inhibition toward the catalytic domain (27), a view consistent with our observed AdoMet-induced structural change. By contrast, the presence of AdoMet significantly protected the regulatory domain-alone hCBS-RD_Δ516–525_ from thermolysin (Fig. 3*C*, *bottom*) (*k_p_*_,+AdoMet_/*k_p_*_,−AdoMet_ ∼0.2), agreeing with its binding to this standalone domain. The substrate serine only appeared to have a significantly protective effect on hCBS-CD (*k_p_*_,+serine_/*k_p_*_,−serine_ ∼0.2). However, on closer inspection, it appeared that a lower molecular mass band of ∼55 kDa was being protected by serine for hCBS-FL (Fig. 3*C*, *asterisk*). This fragment is ∼10 kDa larger than the catalytic domain alone (∼45 kDa), suggesting that serine is protecting some segment of the regulatory domain from proteolysis and that serine binding influences the enzyme beyond its catalytic domain.

##### Ion Mobility Mass Spectrometry Demonstrates Ligand-dependent Conformational Populations

**FIGURE 4.**
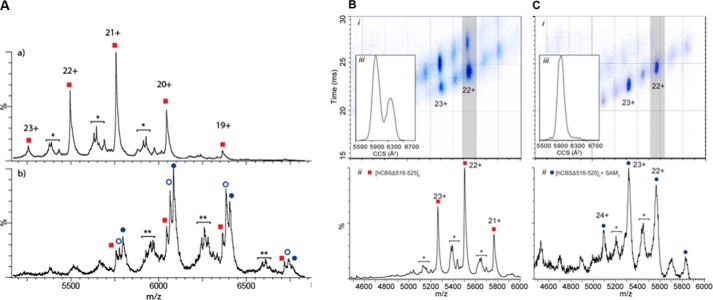
**Binding of AdoMet to dimeric hCBS-FL_Δ516–525_ revealed by mass spectrometry.** *A*, *top*, MS of as-purified hCBS-FL_Δ516–525_ dimer shows a distribution of charge states corresponding to dimeric hCBS (*square*). *Bottom*, mass spectra corresponding to the dimeric hCBS-FL_Δ516–525_·AdoMet complex shows multiple complexes bound to one (*open circle*) and two (*closed circle*) AdoMet molecules. A small abundance of dimers with C terminus deletion (*) and their AdoMet-bound complexes (**) are shown. *B* and *C*, IM-MS spectra were obtained for hCBS-FL_Δ516–525_ in the absence (*B*) and presence (*C*) of AdoMet. *Top*, a plot of *m*/*z* against drift time data. *Bottom*, the mass spectrum charge state series corresponding to dimeric hCBS-FL_Δ516–525_ with minor peaks corresponding to dimers with partial degradation (*). *Inset*, corresponding arrival time distribution for the 22^+^ charge state of hCBS-FL_Δ516–525_. *CCS*, collision cross-section.

We applied mass spectrometry to further investigate the binding of AdoMet to hCBS in solution. First, native MS of the intact complex between hCBS-FL_Δ516–525_ and AdoMet (Fig. 4*A*) revealed an AdoMet stoichiometry of two per hCBS-FL_Δ516–525_ homodimer (*i.e.* one AdoMet per regulatory domain), which supports our structural observations. We next probed the conformational change of hCBS upon AdoMet binding using IM-MS, a method that determines the size and shape of ions based on their ability to traverse a gas-filled mobility cell under a weak electric field. IM-MS data show that hCBS-FL_Δ516–525_ in the as-purified form exhibits two peaks in arrival time distribution (Fig. 4*B*). Because the two peaks are well resolved, they suggest a well defined difference between two discrete states rather than a continuum of interconverting populations. The two discrete states likely represent a mixture of AdoMet-free and AdoMet-bound hCBS in the as-purified sample. This is observed by native MS showing low amounts of AdoMet present (data not shown), possibly carried over from the expression host during purification. Although we cannot rule out the existence of additional conformations or the potential for perturbation of hCBS structure in the gas phase, a conformational ensemble is likely to exist, with a bimodal distribution across drift time. By contrast, hCBS-FL_Δ516–525_ in the presence of AdoMet exhibits essentially one narrow arrival time distribution, suggesting predominantly one conformation (Fig. 4*C*). The results indicate that AdoMet directs the protein into a more uniform conformation.

##### Importance of AdoMet Binding Residues

**FIGURE 5.**
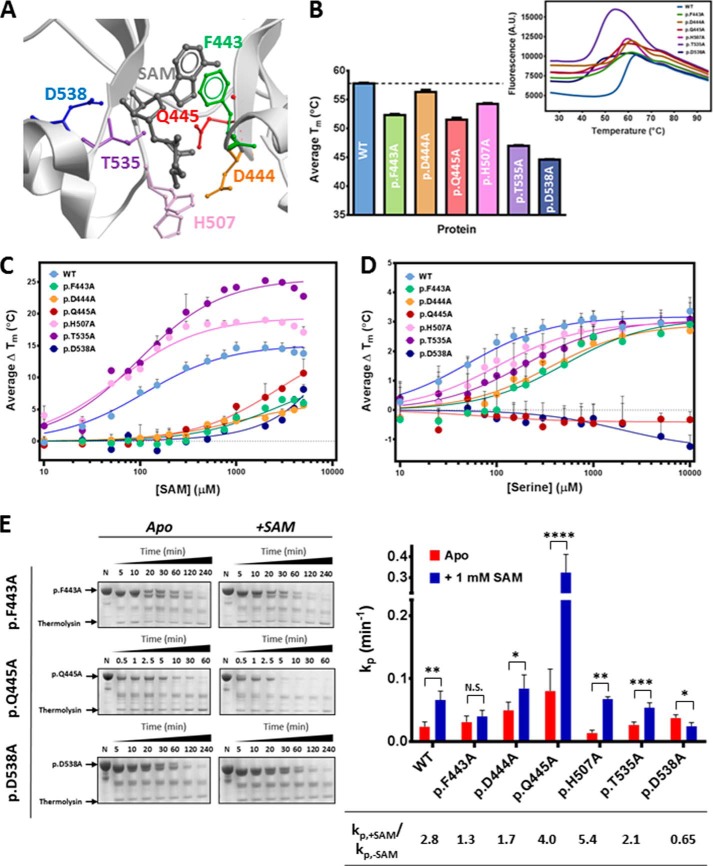
**Delineating the role of AdoMet (*SAM*) binding residues.** *A*, location of each alanine-substituted residue in the hCBS-RD_Δ516–525_ structure. *B*, thermal stability of each alanine-substituted hCBS-FL mutant as compared with wild-type, in the as-purified form. *Inset*, representative thermal unfolding curves. *a.u.*, arbitrary units. *C*, DSF dose-response curves of AdoMet binding for WT and the six alanine-substituted mutants. *D*, DSF dose-response curves of serine binding for WT and the six alanine-substituted mutants. *E*, thermolysin-limited proteolysis for hCBS-FL alanine-substituted mutants to understand their impacts on the conformational response to AdoMet binding. *Left*, representative gels of each assay are shown for p.F443A, p.Q445A (more prone to proteolysis and significantly responded to AdoMet), and p.D538A (protected by AdoMet). *Right*, rate of proteolysis in the AdoMet-free (*red*) and AdoMet-bound (*blue*) forms for hCBS-FL WT and six mutants, showing the abolished proteolysis rate enhancement for p.F443A and p.D538A in the presence of AdoMet. Rates of proteolysis are reported as the means and standard deviations from at least three independent experiments. *p* values were determined using two-tailed unpaired *t* test. *N.S.* = non-significant, *, *p* < 0.05, **, *p* < 0.01, ***, *p* < 0.001, ****, *p* < 0.0001.

Our AdoMet-bound hCBS-RD_Δ516–525_ structure revealed a number of residues involved in ligand binding to the S2 site (Fig. 5*A*). To probe their importance in solution, we performed alanine substitution on Phe^443^, Asp^444^, and Gln^445^ from the FDQ motif, Thr^535^ and Asp^538^ from the GVVTAID motif, as well as His^507^ that is near the S2 site but not directly involved in binding AdoMet, as control. Mutagenesis was performed within the context of our hCBS-FL construct, and all mutants behaved similarly to WT hCBS-FL with regard to soluble expression, heme saturation, and oligomeric status (data not shown).

DSF was then used to interrogate the capacity of the alanine mutants to bind AdoMet, as shown with various hCBS constructs. We initially compared the *T_m_* of WT hCBS-FL with the six mutants in their as-purified forms (Fig. 5*B*) and observed >5 °C reduction in *T_m_* for p.F443A, p.Q445A, p.T535A, and p.D538A as compared with WT, whereas p.D444A and p.H507A have near-WT *T_m_* values. This suggests that Phe^443^, Gln^445^, Thr^535^, and Asp^538^ confer structural stability to the regulatory domain. Indeed, mutating the same set of residues within the context of our hCBS-RD_Δ516–525_ construct resulted in recombinant soluble protein for only p.D444A and p.H507A (data not shown). The *T_m_* difference between the absence and presence of AdoMet (Δ*T_m_*max) was then measured for all proteins (Fig. 5*C*); p.F443A, p.D444A, p.Q445A, and p.D538A all exhibited a much reduced capacity to bind AdoMet, as reflected by their 10–20-fold higher half-maximal effective ligand concentration (AC_50_) (for p.F443A, p.D444A, and p.Q445A) coupled with a reduced Δ*T_m_*max (for p.F443A and p.D444A) (Table 3). Among them, p.D538A showed the lowest response to AdoMet, resulting in an inability to accurately determine the AC_50_ and Δ*T_m_*max values. On the other hand, p.H507A and p.T535A mutants behaved in a similar manner as WT, showing comparable AC_50_ but higher Δ*T_m_*max (likely due to their lower thermal stability in the AdoMet-free form). Overall this demonstrates the important role of Phe^443^, Asp^444^, Gln^445^, and Asp^538^ in binding AdoMet.

**TABLE 3:** **Apparent AC_50_ and Δ*T_m_*max values of hCBS-FL WT and mutant proteins** ND, not determined.

hCBS-FL	Serine		AdoMet	
	Apparent AC_50_	Apparent Δ*T_m_*max	Apparent AC_50_	Apparent Δ*T_m_*max
	μ*m*	°*C*	μ*m*	°*C*
WT	50.7 ± 6.7	3.2 ± 0.1	110.4 ± 13.6	15.1 ± 0.4
p.F443A	504.3 ± 142.5	3.1 ± 0.3	2738 ± 1164	10.8 ± 2.2
p.D444A	356.0 ± 83.6	2.9 ± 0.2	1027 ± 203	6.3 ± 0.4
p.Q445A	N.D.	N.D.	2772 ± 626.6	16.5 ± 1.9
p.H507A	101.2 ± 14.6	3.0 ± 0.1	59.2 ± 8.8	19.4 ± 0.5
p.T535A	190.7 ± 18.6	3.1 ± 0.1	96.4 ± 12.1	25.5 ± 0.7
p.D538A	ND	ND	ND	ND

Our finding that the substrate serine also elicits a *T_m_* shift for WT hCBS-FL and hCBS-FL_Δ516–525_ (Fig. 3*B*) prompted us to test the various mutants in terms of serine response; p.F443A, p.D444A, p.H507A, and p.T535A all demonstrated similar Δ*T_m_*max, albeit with slightly higher AC_50_ values, as WT hCBS-FL (Fig. 5*D*, Table 2), indicating an unchanged response to serine. By contrast, p.Q445A showed no thermal shift, whereas p.D538A becomes slightly destabilized toward serine, suggesting that these two mutants alter the communication from the catalytic to the regulatory domain. These data further support our hypothesis that the regulatory and catalytic domains act in tandem via ligand-induced communication.

Finally, the response of each mutant to AdoMet binding was studied by thermolysin-limited proteolysis (Fig. 5*E*, *left*). The AdoMet-induced proteolytic rate increase for WT hCBS-FL is observed for p.D444A, p.T535A, p.Q445A, and p.H507A mutants (in increasing order of rate enhancement; Fig. 5*E*, *right*), but not for p.F443A. In the case of p.D538A, the addition of AdoMet decreased the proteolysis rate. As a result, the F443A and D538A substitutions did not yield the proteolysis-susceptible conformation associated with AdoMet binding, as shown in WT. Our data therefore agree with the view that Asp^538^ and Phe^443^ are integral to the conformational change associated with AdoMet binding, allowing the transmission of the “AdoMet-bound” signal to the catalytic domain.

## DISCUSSION

The structural basis of hCBS activation has been an area of intensive research over the past years with many postulations about how the regulatory domain inhibits the catalytic domain, and the possible conformational rearrangement associated with AdoMet binding (7, 27, 33, 34). The initial structure of the human dimeric catalytic domain gave insight into the heme and PLP binding mode that is essential for hCBS activity (2). Furthermore the dimeric dmCBS structure revealed a possible activated conformation of the enzyme due to the higher basal activity and AdoMet non-responsiveness of this orthologue. This structure showed the interaction of the two regulatory domains within the homodimer along its two-fold symmetry axis, but did not satisfactorily explain how the human enzyme could be inhibited by these domains and positively regulated by AdoMet (13). The structure of an engineered version of full-length hCBS dimer (14), also determined at a higher resolution in this study, provides a structural interpretation for the lower basal activity of hCBS; the regulatory domains are not interacting at the dimer interface, as found in dmCBS, but are crossed over to contact the catalytic domain of its opposing subunit within the dimer, thereby sterically hindering the active site entrance (Fig. 1*B*). Nevertheless, the binding mode and effect of AdoMet on hCBS were not addressed in these AdoMet-free structures. In this study, we present structural and biophysical data on AdoMet binding to hCBS that uncover four key features.

### 

#### 

##### The Isolated, AdoMet-bound Regulatory Domain Packs as a Dimer

**FIGURE 6.**
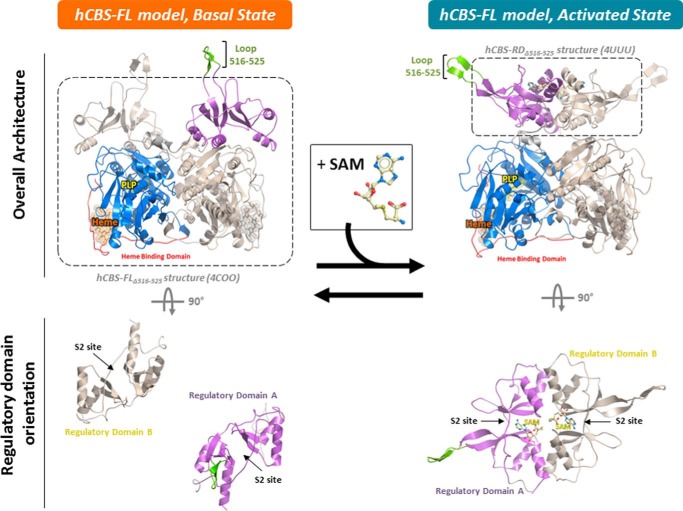
**Postulated mode of AdoMet (*SAM*) activation of hCBS.** In the basal, AdoMet-free state, the regulatory domains within a homodimer do not interact with each other, but with the active site loops of catalytic domains in a domain-swapped manner. This arrangement of regulatory domains results in a low basal activity for hCBS due to the steric hindrance of the regulatory domains on the catalytic domains. AdoMet binding relieves the steric hindrance due to the “un-swapping” and dimerization of the regulatory domains, allowing unhindered access of substrates into the active sites. The hCBS-FL model (with aa 516–525 intact) is generated by SWISS-MODEL homology modeling using structures of hCBS-FL_Δ516–525_ and hCBS-RD_Δ516–525_ (*dashed lines*, this study) templated onto the structure of dmCBS-FL activated state (PDB 3PC2). Domain colors are as of Fig. 1*A* with the additional modeled loop aa 516–525 as *green*. AdoMet is represented as *yellow sticks*.

This arrangement overlays well with the architecture of the regulatory domain of the dmCBS dimer, where minor structural differences are restricted to the linker region connecting the catalytic and regulatory domains. The linker region has some intrinsic flexibility and is consistent with the partial lack of electron density in our hCBS-FL_Δ516–525_ structure. This suggests that the activated state of hCBS could adopt a structural conformation similar to that of the dmCBS basal state, as proposed previously (13, 31), and is supported by following two observations. First, AdoMet binding to the regulatory domain causes a conformational change that is predicted to alter its interactions with the catalytic domain; second, AdoMet binding results in the formation of a dimer as the protein-ligand interactions occur across the interface of the two regulatory domains (Fig. 1*C*). To achieve this, the enzyme therefore has to transition from a basal state as shown in our domain-swapped hCBS-FL_Δ516–525_ structure where the two regulatory domains do not interact, into an activated state where they associate (Fig. 6), akin to the architecture of the dmCBS dimer. This is also consistent with the proposed mechanism from the recently published AdoMet-bound hCBS-FL_Δ516–525,E201S_ data (18), where in the presence of AdoMet, full-length hCBS is no longer domain-swapped with respect to the regulatory domain.

##### AdoMet Binds Only to the Regulatory Domain to Enable Inter-domain Communication

We postulate that the structural rearrangement within the regulatory domain modules, observed upon AdoMet binding, represents part of a communication process transmitted to the catalytic domain. This could disrupt a number of interactions formed at the basal state between the regulatory domain and the active site loop of the catalytic domain (as shown in hCBS-FL_Δ516–525_). An example of how AdoMet binding disrupts inter-domain interactions is provided by the disease-associated S466L mutation in the regulatory domain, which renders the hCBS enzyme constitutively active while retaining the ability to bind AdoMet (6). Inspection of our hCBS-FL_Δ516–525_ structure suggests that the Leu substitution on Ser^466^ may lose its three hydrogen bonds with residues in the active site loop (Fig. 1*C*). As a result, the active site loop could adopt a more dynamic, sequestered conformation, similar to that in the hCBS catalytic domain structure (2). Together, we propose that a rearrangement of the catalytic-regulatory domain organization serves to relieve the steric inhibition imposed by the regulatory domain upon the enzyme active site and to initiate the conformational transition into the enzyme-activated state (Fig. 6). Our ion mobility experiments support this hypothesis, showing that hCBS results in a uniform conformation in the presence of AdoMet (Fig. 4). It is possible that hCBS exists in a dynamic equilibrium between the basal and activated states, which is shifted predominately toward the activated state in the presence of AdoMet. This warrants investigation in future studies.

There is an additional level of communication from the catalytic domain to the regulatory domain, mediated by the substrate serine in the basal state, which alters the stability of the regulatory domain as shown in thermal shift and proteolysis data (Fig. 3). Precedence for such communication comes from studies where missense mutations in the catalytic domain can alter the stability of the regulatory domain (16), likely via the active site loops (aa 170–175, 191–203) that contact the regulatory domain. The various structures of dmCBS and of the activated hCBS catalytic domain all reveal highly dynamic active site loops that close toward the active site in the presence of substrates (2, 13). It is likely that serine binding in the human enzyme also causes conformational changes to the active site loops, similar to those observed in dmCBS. Based on the position of the regulatory domain over the active site loops in the non-activated hCBS-FL_Δ516–525_ structure, these loop conformations may play a role in changing the interface with the regulatory domain. Therefore, in the basal state, the regulatory domain not only sterically blocks the active site, but may also hinder the flexibility of active site loops.

##### One AdoMet Is Bound per Monomer at the S2 Site of the Regulatory Domain

This gives rise to a stoichiometry four AdoMet per tetramer that agrees with previous determination by many techniques over two decades (7, 35, 36), but contrasts with recent findings of possibly two binding sites (S1 and S2) per regulatory domain, reaching as many as six bound AdoMet molecules per hCBS tetramer (16). It had been proposed that the S1 site would also bind AdoMet, but not until the S2 site is pre-occupied (10). Comparison of the AdoMet-free *versus* AdoMet-bound regulatory domain does show a conformational change in the S1 site upon S2 site binding, although no AdoMet was detected in the S1 site (Fig. 2*E*). Native MS of hCBS-FL_Δ516–525_ also confirmed the stoichiometry of one AdoMet per hCBS monomer (Fig. 4). Therefore, at least two possible explanations exist for the discrepancy in stoichiometry data. First, hCBS oligomerization into tetramer or higher order species plays a role in AdoMet binding, such that additional AdoMet binding site(s) could be formed by these intermolecular interactions (*e.g.* AdoMet binding outside of the S2 and S1 sites is known for other CBS domains (30)). Second, the previously reported isothermal titration calorimetry data may be alternatively interpreted (*e.g.* negative cooperativity (32, 37), where AdoMet binding to the first S2 site decreases its affinity to other S2 sites present in the hCBS oligomer, such that stoichiometry may depend on AdoMet concentration). Further work is warranted to answer this ambiguity.

##### Our High Resolution Structures Unequivocally Determine Residues in the AdoMet Binding Pocket

Our hCBS-RD_Δ516–525_ structure not only agrees with the AdoMet binding pocket and stoichiometry as depicted in the hCBS-FL_Δ516–525,E201S_ structure reported at medium resolution (18), but goes further in revealing atomic details of AdoMet binding residues, *e.g.* the involvement of Asp^444^ from both subunits of the dimeric regulatory domain in interacting with AdoMet. Additionally, we probed the importance of these residues, as predicted from crystallography, by alanine mutagenesis in the context of full-length hCBS (Fig. 5). Both thermal shift and limited proteolysis confirmed the essentiality of Phe^443^, Asp^444^, Gln^445^, and Asp^538^ in AdoMet binding. Asp^538^ is conserved in nearly all CBS domain-containing proteins (being part of the GVVTAID signature motif), as is its bifurcated interaction with the AdoMet ribose hydroxyl groups (17). The FDQ motif, by contrast, represents a sequence region fairly unique to CBS enzymes (*i.e.* not commonly observed in other CBS domain-containing proteins), and hence may be endowed with a CBS-specific role in AdoMet-activated response. To this end, among the four essential AdoMet binding residues, Phe^443^ and Asp^538^ play a further role in inter-domain communication, as suggested by an altered response to AdoMet by the p.F443A and p.D538A mutants in DSF studies. This further suggests that conservation of the AdoMet binding mode is important for the regulatory role of this domain across the polypeptide in the catalytic domain.

In conclusion, we report here high-resolution structures of hCBS without and with AdoMet bound, giving insight into its activation by this modulator. The structural similarity to the arrangement of the dmCBS regulatory domains leads us to propose that the activated conformation is similar to that of dmCBS. Additionally, our solution studies have given further insight into the inhibitory mode of action of the regulatory domain in the basal state. Alanine mutagenesis studies also confirmed the importance of a number of residues interacting with AdoMet and suggest that these will be important in the rational design of small molecule modulators of hCBS activity.

## Footnotes

3 The abbreviations used are:

CBS: cystathionine β-synthase
hCBS: human CBS
dmCBS: *Drosophila melanogaster* CBS orthologue
PLP: pyridoxal 5′ phosphate
AdoMet: *S*-adenosyl-l-methionine
DSF: differential scanning fluorometry
IM: ion mobility
r.m.s.d.: root mean square deviation
aa: amino acids
FL: full-length
RD: regulatory domain
CD: catalytic domain.
